# Solvent Quality and Aggregation State of Asphaltenes on Interfacial Mechanics and Jamming Behavior at the Oil/Water Interface

**DOI:** 10.1021/acs.langmuir.3c01890

**Published:** 2023-10-20

**Authors:** Junchi Ma, Olivia M. Haider, Chih-Cheng Chang, Kathryn A. Grzesiak, Todd M. Squires, Lynn M. Walker

**Affiliations:** † Department of Chemical Engineering, Carnegie Mellon University, Pittsburgh, Pennsylvania 15213, United States; ‡ Department of Chemical Engineering, University of California, Santa Barbara, California 93106, United States; § The Dow Chemical Company, Midland, Michigan 48640, United States

## Abstract

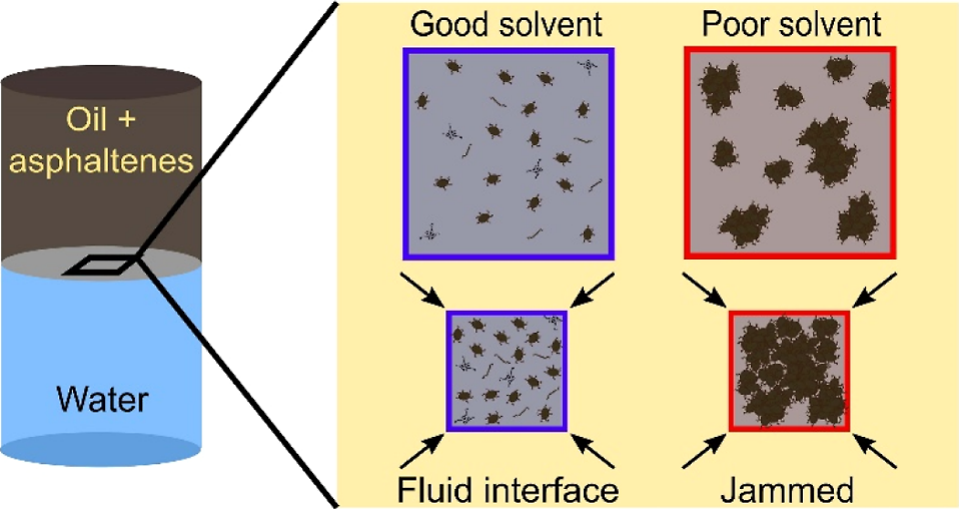

The formation of highly stable water-in-oil emulsions results in complications in both upstream and downstream processing. Emulsion stability in these systems has been connected to the adsorption of surface-active asphaltenes that are assumed to form a rigidified film at the oil/water (o/w) interface. Full characterization of this behavior is needed to allow for engineered solutions for enhanced oil recovery. Interfacial properties, such as surface pressure and interfacial elasticity, are implicated in the stabilizing mechanism for these observed films. Asphaltenes are known to be interfacially active in both good solvents (aromatics) and poor solvents (high ratio of aliphatic to aromatic). However, due to inherent complexities present in asphaltene studies, the details of the mechanical properties of the interface remain poorly understood. Despite the widely accepted perception that asphaltenes form persistent rigid films at fluid–fluid interfaces, the connection between bulk solution properties and interfacial mechanics has not been resolved. Here, the effects of solvent quality on the interfacial properties of asphaltene dispersions are determined by using a well-defined asphaltene/solvent system. Interfacial rigidity is observed only under poor solvent conditions, while the good solvent system remains fluid-like. The interfacial rheology under good and poor solvent conditions is measured simultaneously with surface pressure measurements to track interfacial development. It is shown that surface pressure and dilatational modulus measurements are not indicators of whether an interface demonstrates rigid behavior under large compressions. Finally, conditions required for asphaltene-coated interfaces to exhibit the mechanical behavior associated with a rigidified interface are defined. This work provides a framework for quantifying the impact of the aggregation state of asphaltenes on the stability and mechanics at the o/w interface.

## Introduction

Asphaltenes are a class of polyaromatic molecules naturally existing in crude oil^1,2^ and are defined as the fraction of crude soluble in aromatics and insoluble in alkane solvents.^3−5^ Colloidal behavior of asphaltenes, as characterized in the Yen–Mullins model, shows that even in well-dispersed, “good” solvent systems, asphaltenes undergo irrepressible association into nanoaggregates (typically ∼2 nm) and clusters (∼5 nm).^1,5^ These aggregates are colloidally stable and the association is shown to have a concentration dependence.^6−8^ The solvent quality controls dispersibility and solubility of bulk asphaltenes.^1−3,9^ Solubility profiles of different asphaltene samples, defined with respect to a given *n*-alkane, have been characterized in the bulk as a function of the heptane-to-toluene ratio.^3,10−12^ From these studies, it was determined that the onset of asphaltene precipitation begins at a toluene fraction of 40% or less by volume.^3,13^ As the aliphatic volume fraction is reduced, the solvent quality transitions to good solvent conditions, where asphaltenes exhibit greater long-term stability. The connection between aggregation and precipitation is not well understood nor is the relative surface activity of different aggregate fractions.

Asphaltenes are interfacially active in good and poor solvents, and solvent quality is known to impact the characteristic adsorption time scales^14,15^ and mechanical properties of the oil/water (o/w) interface.^16−18^ When dispersed in good solvents, meaning asphaltenes are well-dispersed and colloidally stable, the interfacial activity of asphaltenes is lower compared to asphaltenes in poor solvent environments, evidenced by interfacial tension dynamics and the steady-state surface pressure.^19,20^ Asphaltene-laden o/w interfaces have been shown to form mechanically rigid films,^14,21−23^ observed qualitatively through buckling under large interfacial compressions and deformations. This rigidity has been associated with the formation of highly stable water-in-oil emulsions^5,24−26^ leading to equipment fouling and decreased efficiency in the extraction and processing of crude oils.^5,27−30^ The mechanism responsible for interfacial stabilization and the mechanics of these observed rigid films need to be fully understood to control and predict coalescence of these water-in-oil emulsions in crude oil. These quantitative characterizations are needed as a framework to design demulsifiers and other processing aids.

Many interfacial studies have been conducted to characterize the properties of asphaltenes at the o/w interface. The soft-glassy rheology model has been used to characterize the structure of an adsorbed asphaltene layer, exhibiting a soft-glassy behavior with yield stress.^31,32^ Local inhomogeneities at the interface due to the formation of asphaltene aggregates are shown by interfacial shear measurements via the microbutton technique.^33^ Molecular simulations at the o/w interface have been used to further understand the effects of asphaltene structure, solubility, surface affinity, and orientation at the interface on interfacial stability.^34−36^ Small-angle neutron scattering measurements have been performed on the adsorbed layers of asphaltenes for the water-in-oil emulsions, and the radius of gyration of the asphaltene aggregates/clusters was measured as a function of ionic strength, resin content, and pH.^37−39^ Larger interfacial aggregate sizes, as well as an increase in emulsion stability, were observed in systems with lower resin quantity and increased ionic strength. Furthermore, as the pH of the systems increased (>7), emulsion droplets demonstrated a decrease in the surface area-to-volume ratio as well as an increase in both interfacial thickness and size of the adsorbed aggregates. The impact of the solvent environment is a key parameter in determining the mechanics of the interface. The stability of the emulsions containing the asphaltene layer on the interface has been studied as a function of aromatic content in the solvent, illustrating that higher aromatic content leads to decreased emulsion stability resulting in increased water resolution in crude oil processing.^17,37,40−43^ Systems with relatively high aliphatic compositions destabilize bulk asphaltenes, leading to precipitation and changes in bulk asphaltene concentration during the interfacial measurements.^44^ In these poor solvent conditions, lower interfacial elasticity has been observed in both dilatational and shear rheology measurements^45−47^ and has been inferred as a decrease in the stability of the o/w interfaces.^48^

Two major challenges that make direct comparisons between asphaltene studies difficult are variations in the molecular makeup of asphaltene samples and inconsistencies in the experimental conditions used across individual studies. Asphaltenes do not share a single, discrete chemical structure; molecular compositions vary depending on the geographical location of the crude oil source, solvents used for precipitation from the bulk, and the specific batch of extracted asphaltenes.^49,50^ Chemical properties, like the number of heteroatoms such as sulfur, iron, and nitrogen, vary between samples from different reservoirs.^51−54^ The number of aromatic rings and overall chemical composition of the molecules will also differ within the same asphaltene sample.^3,30,49,52−55^ At the o/w interface, asphaltenes from different sources have been shown to demonstrate differences in both interfacial coverage^56^ and interfacial shear moduli^47^ under the same solvent conditions. Thus, due to the inherently different molecular compositions between asphaltene samples, results from interfacial studies on asphaltenes are often inconsistent.

Additionally, as asphaltenes are defined by solubility properties, the type of solvent or dispersant changes the solubility and interfacial activity of asphaltene molecules. Different model systems are often reported in the literature and will vary not only in the asphaltene source of crude oil^14,21,57^ or dilute bitumen^22,58,59^ but also in the ratio of aromatic and aliphatic solvents used to disperse the asphaltene samples. Typically, asphaltene studies use a mixture of heptane and toluene (referred to as “Heptol”)^60^ or pure toluene.^61,62^ These solvents have certain drawbacks for studying asphaltene-coated o/w interfaces.^22,63^ Both heptane and toluene are volatile, and the solubility of these oils in water leads to mutual partitioning at the o/w interface. Spontaneously formed emulsions of water droplets in toluene have been observed due to the enhanced partitioning of water in toluene by asphaltenes.^61,62^ This partitioning can also cause the interface to be unstable due to the induced flow introducing large noise for the interfacial characterizations.^64^ A comprehensive understanding of the interfacial behavior of asphaltenes at o/w interfaces is limited by these inconsistencies in both the asphaltene sample and the solvent system used in the literature. Thus, it is crucial to compare the interfacial measurements on a single asphaltene sample in a well-defined dispersant system.

The goal of this work is to fully characterize the interfacial properties of asphaltene subfractions when dispersed in aromatic- and aliphatic-rich solvent environments. Using a millifluidic approach detailed in a previous publication,^65^ the long-term stability and precipitation behavior of asphaltenes dispersed in mixtures of Isopar M and Aromatic 200 were determined and used to define “good” and “poor” solvent conditions for interfacial measurements. Aggregation in the bulk caused by poor solvent conditions leads to changes in bulk asphaltene structure and, by extension, interfacial properties of the o/w interface. Using interfacial studies under good and poor solvent conditions, the conditions needed to analyze and characterize interfacial properties in systems with enhanced asphaltene aggregation will be determined. Here, the dynamic adsorption of asphaltenes and the simultaneous evolution of the interfacial mechanics are measured in each solvent environment. The interfacial shear and dilatational rheology are compared for interfaces in systems where asphaltenes are considered to be dispersed in good solvent conditions to set up as a base case, where asphaltenes are colloidally stable over long experimental times. Although different techniques are used to characterize these interfacial properties, the interfaces have comparable length scales and experimental conditions.

## Experimental Section

Two asphaltene samples, denoted as C5 and C7 in this work, were prepared by Dow Chemical Company (Midland, MI). Both subfractions were extracted from naphtha-diluted bitumen samples from the same geographical source in Alberta, Canada. The samples were first purified to remove the diluent, and then a spinning band distillation with 18 theoretical plates and a reflux ratio of 25:1 separates the bitumen from the solvent. The purified bitumen includes all fractions boiling above 200 °C. The asphaltene subfraction C7 in this work was prepared by mixing *n*-heptane (Fisher Scientific, Waltham, MA) with 45 g of purified bitumen at a weight ratio of 40:1 in a glass container, while C5 was prepared with *n*-pentane to extract the asphaltenes from the purified bitumen. The mixture was shaken until the bitumen was homogeneously mixed into the solvent. The mixture was allowed to sit for 48 h at approximately 20 °C to allow solids to settle out of suspension. Approximately 60% of the solvent was decanted, then the remaining solvent and precipitated solids were transferred to 1 L Nalgene high-density polyethylene bottles and centrifuged at 3000 rpm for 10 min. The solids were washed two more times by decanting the supernatant, replacing it with fresh *n*-heptane (C7) or *n*-pentane (C5) to disperse the solids again, and then repeating the centrifugation. After the supernatant was decanted one last time, the precipitate was dried in a vacuum oven at 60 °C to remove residual solvent. The solvents Isopar M (ρ = 0.784 g/mL) and Aromatic 200 (ρ = 0.993 g/mL) were provided by ExxonMobil Chemical Co. (Spring, TX) and used as received. Isopar M is a branched alkane solvent with a carbon number of *n* = 11, and Aromatic 200 is an aromatic solvent composed primarily of naphtha and methylnaphthalene. Compared to the “Heptol” solvent systems often used for interfacial asphaltene studies, both oils used in this work are considerably less volatile (vapor pressure of approximately 10 Pa at room temperature) and have lower solubility of water into the oils, and oils into water, to avoid mutual partitioning.^64^

A ratio of high aromatic solvent, consisting of 20% Isopar M and 80% Aromatic 200 by volume, showed long-term colloidal stability on the order of months^65^ using a millifluidic device appropriate for characterizing highly turbid colloidal systems.^66^ This ratio of 1–4 Isopar M to Aromatic 200, referred to as isomatic 1–4 in this work, is defined as a good solvent and demonstrates no bulk precipitation of C5 or C7 over months at rest under ambient conditions. As the aliphatic volume fraction was increased, the solvent quality transitioned to poor solvent conditions, where asphaltene aggregates were destabilized. At a ratio of 50% Isopar M and 50% Aromatic 200 by volume (isomatic 1–1) asphaltene clusters aggregated further; however, the timescale at which the onset of precipitation observed was longer than the timescale of the interfacial experiments, minimizing changes in bulk concentration during interfacial characterization. Under these conditions, the asphaltene aggregates, larger than the colloidally stable structures defined in the Yen–Mullins model, were present in the bulk solution, and this 1–1 volume ratio was used here as the “poor solvent”. Interfacial tension measurements at the clean o/w interface for isomatic 1–4 and isomatic 1–1 were 41 ± 1 and 44 ± 1 mN/m, respectively. These model systems were used to disperse asphaltene samples for characterization of the interfacial tension and rheology during transient adsorption and interfacial aging.

A pendant drop setup was used to qualitatively observe the effect of solvent composition on interfacial properties. Samples of C5 asphaltenes were dispersed in solvent ratios of 1–4, 1–2.3, 1–1.5, and 1–1 Isopar to Aromatic 200. A drop containing the asphaltene dispersion was pinned at the tip of a borosilicate glass capillary and suspended in a glass cell containing 20 mL of deionized (DI) water with a set resistivity of 18.2 MΩ. The cell has a rectangular cross section of 2 cm × 2.3 cm, and the glass capillaries from World Precision Instruments, Inc. (Sarasota, FL) have an internal diameter of 0.75 mm. Interfaces were aged for 3 h and then subjected to controlled interfacial compressions with ∼90% areal change. Compressions were conducted using an air-line connected to the asphaltene-filled capillary and a programable syringe pump (Braintree Scientific, Inc.) and a 3 mL syringe of diameter 8.66 mm (Becton Dickinson Plastipak). Interfaces were imaged at 25 fps using a camera attached to a Nikon microscope (objective 10×) and recorded using a script written in LabVIEW from National Instruments.

**Figure 1:**
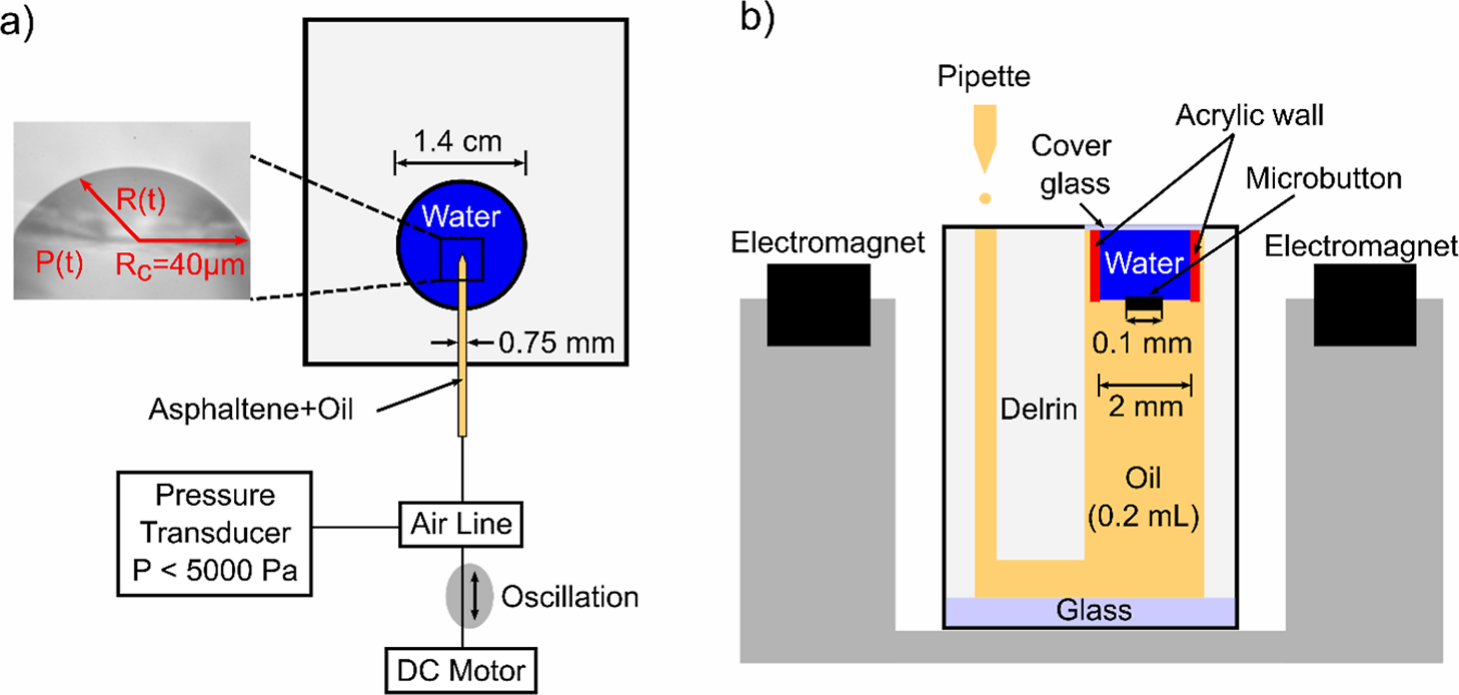
(a) Microtensiometer platform based on a capillary tensiometer with inline pressure transducer and motor to provide oscillatory pressure for dilatation and (b) microbutton device redesigned for water-on-oil experiments (the microbutton and capillary are not to scale).

Measurements of interfacial tension and dilatational rheology were performed using a microtensiometer platform.^67−71^ This platform, shown in Figure 1a, has been used to characterize complex fluid–fluid interfaces in a number of systems^67,69,70,72−75^ and is built based on the principle of a capillary tensiometer. A glass capillary was filled with the oil phase fluid and connected in line with a pressure transducer (Omegadyne Inc., Sunbury Ohio). Glass capillaries, detailed above, were pulled to a tip radius of 36–45 μm using custom settings on a PMP-102 micropipette puller (MicroData Instrument, Inc., South Plainfield, NJ). The interior of the capillary was acid-washed with 30% sulfuric acid in DI water and then coated with hydrophobic material from Dynasylan SIVO CLEAR (Evonik Industries, Essen, Germany) to ensure the three-phase contact line remains pinned at the tip of the capillary. Capillaries were rinsed with ethanol after coating and then baked at 60 °C for 30 min prior to use. The capillary was placed in a 3D-printed cell made from Delrin (polyoxymethylene) and submerged in a semi-infinite reservoir (∼3 mL) of DI water. The o/w interface was imaged on a Nikon T-300 inverted light microscope. The radius of the interface was measured using routines written in LabVIEW (National Instruments). From the measured radius of curvature and pressure jump across the interface, the interfacial tension was determined using the Young–Laplace equation
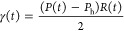
1where *P*(*t*) is the Laplace pressure difference across the interface as a function of time, *P*_h_ is the hydrostatic pressure, *R*(*t*) is the radius of the interface as a function of time, and γ(*t*) is the calculated dynamic interfacial tension. The hydrostatic pressure was determined by measuring the interfacial tension at different values of pressure and radius for a clean o/w interface.

Interfacial dilatational rheology was performed by applying a low-amplitude oscillatory pressure to the interface via the DC motor, shown in Figure 1a, to impose a change in the surface area of the interface. The frequency of the oscillations ranged from 0.5 < ω < 5 rad/s, and the amplitude was constant at 90 Pa. Dilatational modulus is defined as the change in surface excess normal stress *P*^S^ with interfacial area *A*
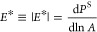
2

In the absence of bulk fluid stresses, *P*^S^ is related to the pressure jump across the interface, Δ*p*
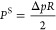
3

The area, *A*, is calculated as the area of a hemispherical cap
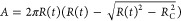
4where *R*_C_ is the radius of the capillary. The measurement of uncertainty of the radial measurement was 0.1 μm, which resulted in the maximum measurable modulus on the order of 1000 mN/m.^73^

Interfacial shear rheology was measured using a ferromagnetic microbutton^33,76^ as shown schematically in Figure 1b. Details of the components of the microbutton are shown in Figure S1 in the Supporting Information. The microbutton (*d* = 0.1 mm) sits on a stable water-on-oil interface. An acrylic capillary combined with a coverslip was used to hold the water phase and microbutton. The setup containing the microbutton and water was placed on a Delrin sample cell, and the oil phase was injected through the side channel until the water and oil were in contact. Four electromagnets were placed around the water–oil sample cell to generate a magnetic field to rotate the microbutton. A bright-field microscope (Eclipse 80i, Nikon) connected to a CCD camera (CV-A10CL, JAI) was applied to visualize the interface. A custom LabVIEW program was used to control the magnetic field, record the microbutton rotation, and measure the interfacial shear rheology. The electromagnets were used to apply a known magnetic field on the microbutton and the angular displacement, Δθ, of the microbutton was measured. The dimensionless Boussinesq number, *Bo*, is defined as
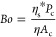
5where *P*_c_ is the perimeter of contact between the probe and interface layer, *A*_c_ is the contact area, η is the viscosity of the bulk fluid, and ηs* is the interfacial viscosity. *Bo* compares the magnitude of the drag force exerted by the interface on a probe relative to the drag force exerted by the bulk fluid. At the limit of high Boussinesq number, *Bo* ≫ 1, the interfacial drag dominates the interface so the interfacial complex viscosity can be calculated
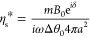
6where *m* is the magnetic moment of the microbutton, *B*_0_ is the amplitude of the magnetic field, ω is the angular frequency, δ is the phase lag between angular displacement and relative torque, and *a* is the radius of the microbutton. The complex viscosity can then be related to the complex modulus, G*, where
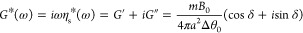
7

Two components, *G*′ and *G*″ are the elastic (in-phase) modulus and viscous (out-of-phase) modulus, respectively. In this paper, the magnitude of the complex modulus |G*| and phase angle δ, represent the interfacial stiffness and viscoelasticity with
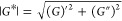
8

To characterize the interface at a larger scale and later stages of asphaltenes adsorption, an AR-G2Magnetic Bearing Rheometer (TA Instruments) with double wall ring (DWR) geometry was also used to measure the interfacial shear rheology. The DWR is a platinum ring with a radius of 35 mm and a square-edged cross section so the ring can pin at the water–oil interface. The upper sensitivity limits of the DWR rheometer and the microbutton were 5 and 0.01 μN·s/m, respectively. With a higher upper detection limit, the DWR was used to characterize the stiffer interfaces. To form a stable oil-on-water interface with the ring pinned at the interface, water is first injected into the Delrin sample cell using a transfer pipette. The edge of the sample cell is pinned at the water surface, so the curvature of the water surface can be adjusted manually. After forming a flat surface of water, the ring was placed on the water surface. Approximately 3 mL of oil was then deposited uniformly from the top of the ring and over the whole sample cell to form a stable water–oil interface.

## Results and Discussion

**Figure 2:**
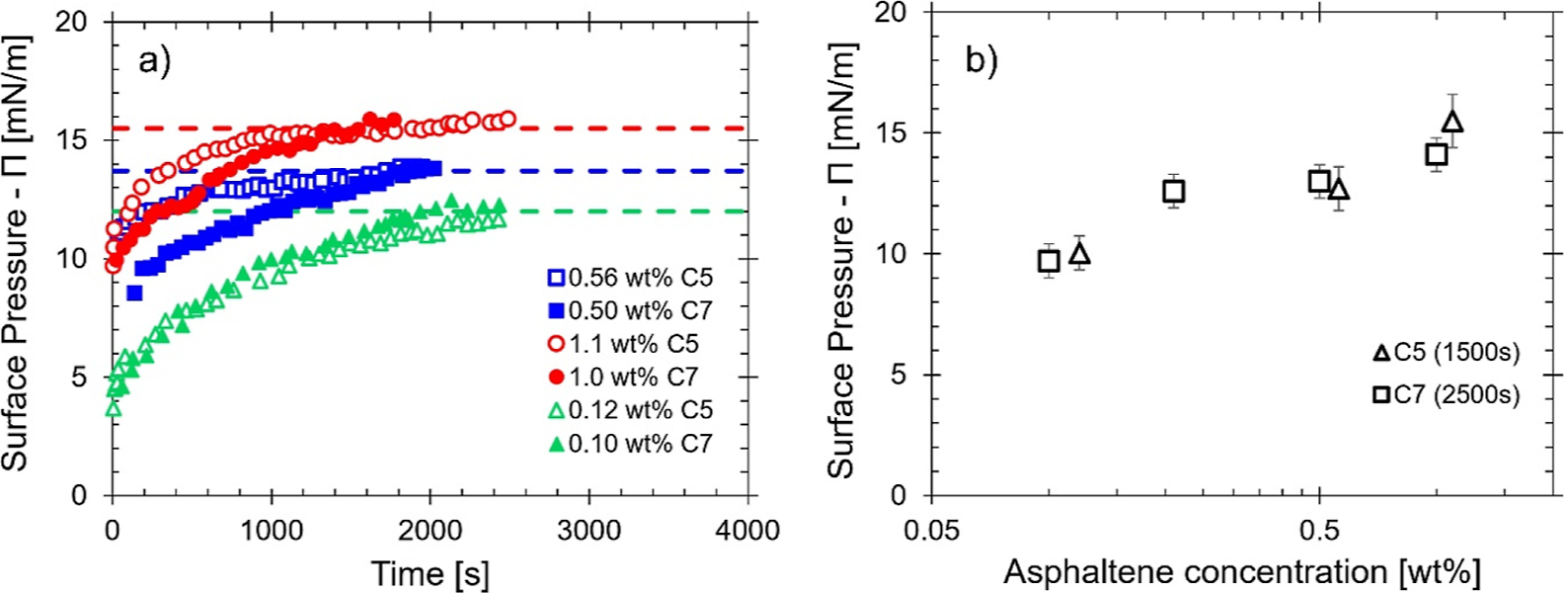
(a) Surface pressure (Π = γ – γ_0_, γ_0_ = 41 ± 1 mN/m) as a function of time for C5 asphaltenes in isomatic 1–4 (open symbols) at concentrations of 0.12 wt % (green triangles), 0.56 wt % (blue squares), and 1.1 wt % (red circles), as well as C7 asphaltenes in the same solvent conditions (closed symbols) at concentrations of 0.1 wt % (green triangles), 0.5 wt % (blue squares), and 1 wt % (red circles). Dotted lines are included to guide the eye. (b) Surface pressure as a function of asphaltene concentration for C7 (squares) at 2500 s and C5 (triangles) at 1500 s.

Because both asphaltene fractions were precipitated from the same geological source, they were expected to be chemically similar, with differences being primarily due to the samples being precipitated out of the bitumen with alkanes of different molecular weights, resulting in different fractions. Figure 2a compares the dynamic measurements of surface pressure, Π = γ – γ_0_, of C5 (open symbols) and C7 (closed symbols) asphaltene samples adsorbing to the o/w interface at different bulk concentrations in the good solvents isomatic 1–4. The colors and symbols are used to highlight systems of similar bulk concentration. For all asphaltene concentrations, *t* = 0 corresponds to the formation of a new, clean o/w interface. Due to rapid initial adsorption to a clean interface, the initial increase in surface pressure is not captured in these measurements. At all concentrations measured, the surface pressure was still changing slightly and did not reach an equilibrium value within at least 2500 s. Here, the system is defined as being at equilibrium when the change in surface pressure is less than 1 mN/m within 1000 s.

From the interfacial dynamics, both C5 and C7 are interfacially active in a good solvent system. Both exhibit a clear trend with an increase in surface pressure during the first 1000 s, followed by a slower increase at longer times. This is consistent with the observations of the two stages of asphaltene adsorption argued in the literature,^14,46^ where there is an initial diffusion-limited adsorption followed by a longer reorganization of the asphaltenes at the interface. Asphaltene concentration influences interfacial dynamics, with a higher bulk concentration having more rapid dynamics and higher surface pressures, as shown in Figure 2a. At comparable bulk concentrations, the surface pressure for both C5 and C7 asphaltenes are shown to approach the same surface pressure at long times (*t* > 1500 s), as shown in Figure 2b. This suggests that for these two samples, the method of fractionation of the asphaltene does not significantly impact the ultimate surface concentration.

**Figure 3:**
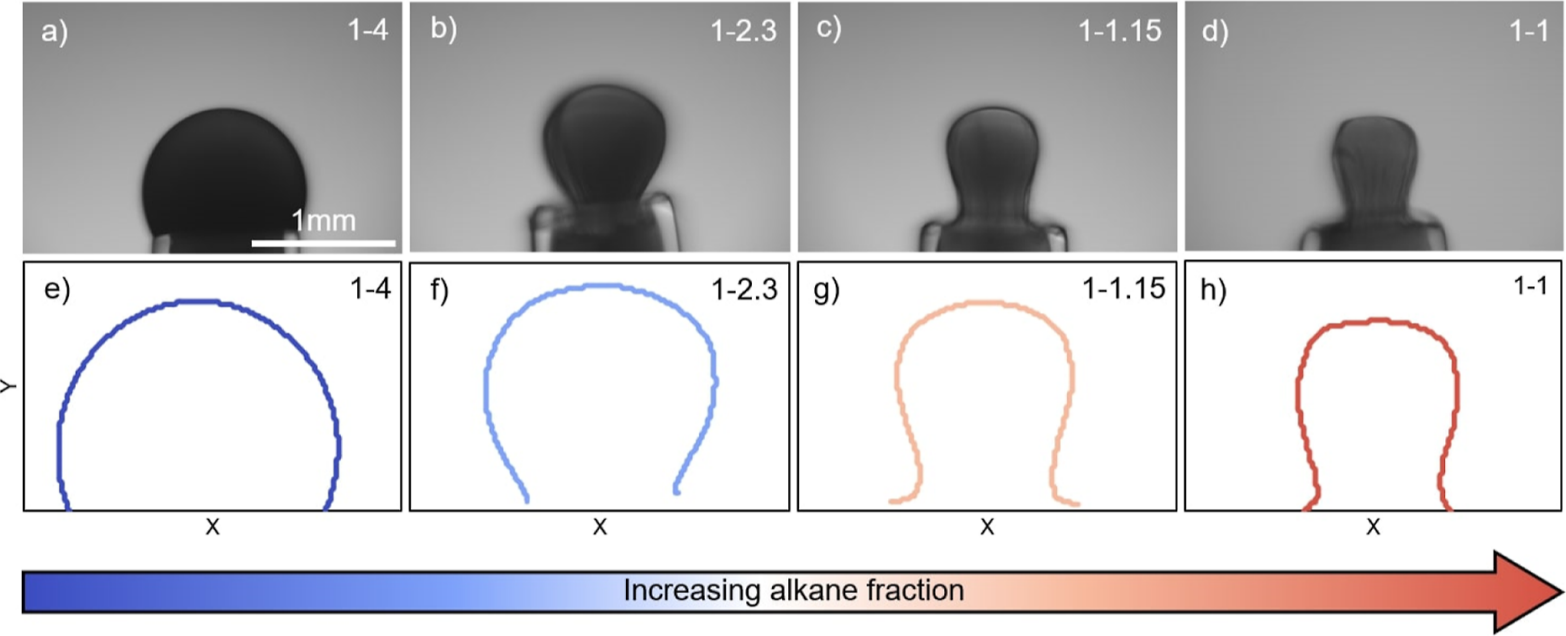
Pendant drop images of asphaltene-laden interfaces after a 3 h interfacial aging time followed by a 90% areal compression for individual drops containing 1 wt % C5 asphaltenes dispersed in mixtures of (a) 1–4, (b) 1–2.3, (c) 1–1.15, and (d) 1–1 Isopar M to Aromatic 200, suspended in a reservoir of DI water. (e–h) Interfacial shape of compressed drops at increasing alkane fraction.

To observe the effect this solvent composition has on the development of rigid interfaces in both good and poor solvents, a qualitative pendant drop study was used to record the interfacial behavior during a large-strain interfacial compression. Figure 3 shows images of drops containing 1 wt % C5 dispersed in isomatic 1–4 (a), a 1–2.3 ratio of Isopar M to Aromatic 200 (b), a 1–1.15 ratio (c), and the poor solvent isomatic 1–1 (d). Drops were dispersed in a reservoir of DI water; individual interfaces were aged for 3 h prior to the compression process. To better show any roughness or nonuniformity of the interface due to rigidity, the edge of the interface was traced using MATLAB software. Figure 3 shows the outline of the drop shapes from tracing the edge of the interface after being subjected to the same large compression for increasing alkane fraction. Under good solvent conditions (isomatic 1–4), the interface is smooth and maintains a spherical shape. As the alkane fraction in the solvent increases (isomatic 1–2.3, 1–1.15, and 1–1), the interface deviates from the spherical shape. At a ratio of 1–1, there is visible buckling of the interface as well as wrinkles at the neck of the drop. Under this poor solvent condition, dispersed asphaltenes are destabilized and aggregate further, forming larger clusters that can flocculate and precipitate out of solution.^52,65,77^ This suggests that the aggregation of asphaltenes, driven by the composition of the solvent environment, significantly impacts the mechanical properties and is a requirement for rigidity.

**Figure 4:**
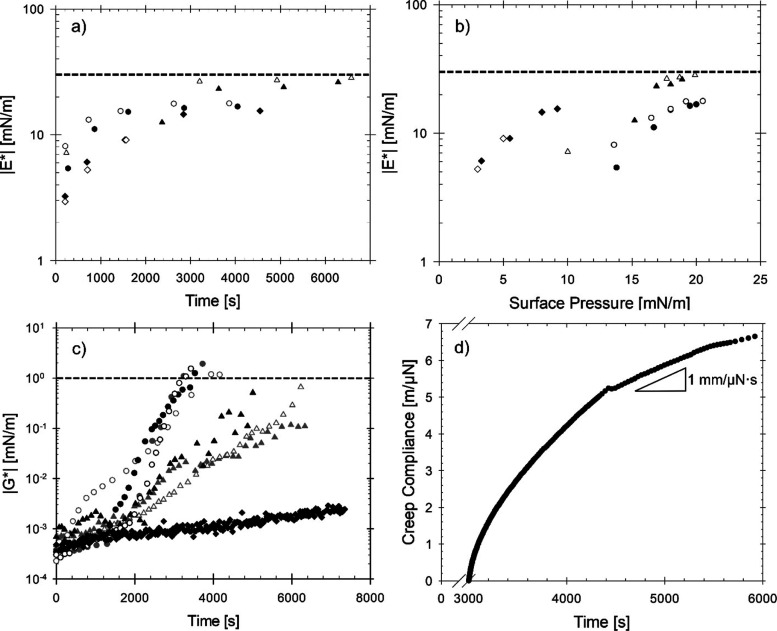
Dilatational modulus, |E*|, measured at a single frequency (ω = 0.54 rad/s) as a function of (a) time and (b) surface pressure for 0.01 wt % (diamonds), 0.1 wt % (triangles), and 0.5 wt % (circles) of asphaltene C7 in isomatic 1–4. Filled and empty symbols represent two trials. A dashed line is drawn at 30 mN/m to show the approximate plateau value of the dilatational modulus. (c) Interfacial shear modulus, |G*|, as a function of time for C7 at bulk concentrations of 0.01 wt % (diamonds), 0.1 wt % (triangles), and 0.5 wt % (circles) measured with a microbutton technique. The dashed line on (c) shows the upper detection limit of the measurement. Three replicates are performed on 0.1 and 0.5 wt % differentiated by different fills. (d) Creep compliance of an aged C7-coated interface (initial age of 3000 s) versus time. Compliance is measured with DWR with 0.5 wt % bulk asphaltene C7 concentration. The applied torque is maintained at 0.005 μN·m.

Mechanical properties of asphaltene-laden interfaces under good solvent conditions were characterized using C7 asphaltenes. Dilatational rheology was performed on the microtensiometer by probing the interfacial mechanics during adsorption and aging of the interface while measuring the dynamic interfacial tension. The dilatational modulus, measured at a single frequency of 0.54 rad/s, is shown in Figure 4a and was recorded over time for samples containing 0.01, 0.1, and 0.5 wt % C7 in isomatic 1–4. At each concentration measured, the magnitude of the dilatational modulus slowly increased with time and reached a plateau value of approximately 30 mN/m after 4000 s. Figure 4 shows the measured dilatational modulus at the same bulk asphaltene concentrations of C7 as a function of the surface pressure. A dotted line is drawn at 30 mN/m, marked as the apparent plateau of the modulus value. For the lowest asphaltene concentration (0.01 wt %), the surface pressure is overall lower than the two higher concentrations, but the dilatational modulus approaches a similar value. The impact of measuring transient dilatational modulus on the interfacial dynamics has been verified to be minimal in this system (see Figure S2 in the Supporting Information) and in previous work.^73^ As the interface ages, the increase in the dilatational modulus is consistent with the adsorption process of C7 from the oils but is not dependent on bulk concentration. These C7-coated interfaces have similar values of dilatational modulus when compared to simple surfactant-laden interfaces^78^ (|E*| ≈ 10 mN/m) and are not high enough to indicate the development of a rigidified interface or significant lateral interaction between adsorbed species. This illustrates how, while increasing bulk concentration increases surface pressure, it has no strong impact on the dilatational modulus at long aging times and does not necessarily induce a higher elasticity of the o/w interfaces at these dilute concentrations.

To compare to the dilatational rheology, Figure 4c shows the dynamics of interfacial shear modulus, |G*|, measured using the microbutton technique at the frequency of 6.3 rad/s for 0.01, 0.1, and 0.5 wt % C7 in isomatic 1–4. For the rheological response of the C7-coated interface on a macroscopic perspective, DWR is used for interfacial shear moduli above the upper limit (1 mN/m) of the microbutton device, shown with the dashed line in Figure 4c. The interfacial shear response is the weakest at 0.01 wt % C7, as the shear modulus increases less than 3 μN/m after 2 h. The highest concentration has the fastest dynamics, reaching the upper detection limit after 3000 s. However, the magnitude of the interfacial shear modulus is on the order of 1 mN/m, again showing no indication of rigidification after aging for 1 h. The creep compliance at a constant torque value of 0.005 μN·m, shown in Figure 4d, demonstrates that the coated interface still behaves as a viscoelastic liquid after 6000 s of aging time. A sample of 0.5 wt % C7 adsorbs from the oils for 3000 s before the creep compliance is measured on the aged o/w interface. The torque used is within the linear viscoelastic region, shown in Figure S3 in the Supporting Information. The long-term slope is 1 mm/μN·s, resulting in an interfacial shear viscosity of 1000 μN·s/mm. In characterizing the shear and dilatational rheology of these asphaltene-laden interfaces, these results consistently demonstrate viscoelastic properties rather than rigidification when asphaltenes are dispersed in a good aromatic-rich solvent environment.

**Figure 5:**
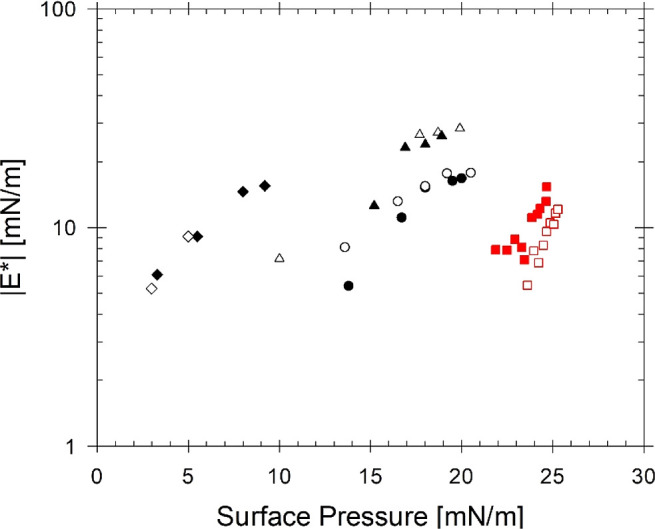
Dilatational modulus, |E*|, measured at a single frequency (ω = 0.54 rad/s) as a function of surface pressure. The black symbols correspond to samples with C7 asphaltenes dispersed in isomatic 1–4 at concentrations of 0.01 wt % (diamonds), 0.1 wt % (triangles), and 0.5 wt % (circles). The red squares correspond to the system with 1 wt % C5 dispersed in isomatic 1–1, which are conditions in which buckling was observed under large strain compressions. Filled and empty symbols represent two replicates under the same condition.

The complicated interfacial behavior associated with the rigidity of asphaltene interfaces is observed as the aliphatic fraction increases, creating poor solvent conditions for dispersed asphaltenes. Figure 5 shows the same dilatational modulus data of C7 in isomatic 1–4 from Figure 4b, plotted against dilatational modulus measurements of 1 wt % C5 in the poor solvent (isomatic 1–1) taken at the same frequency. For the poor solvent conditions, dilatational measurements probe interfacial development as the interface ages for 3 h, corresponding to the time the interfaces age in the macroscopic pendant drop results shown in Figure 3. The dilatational modulus under both solvent conditions is shown to increase with increasing surface pressure and demonstrates that asphaltenes are still surface-active in a poor solvent. While the surface pressures measured for asphaltenes in the poor solvent are higher overall than in the good solvent, the dilatational modulus after the same interfacial aging time is on the same order (∼10 mN/m). As shown in the pendant drop results, the system with 1 wt % C5 asphaltenes in isomatic 1–1, the interface demonstrates the behavior of a rigidified interface with visible buckling after a high-amplitude compression is applied. However, there is no indication of a rigidified interface under these same system conditions from either the surface pressure or small-amplitude dilatational modulus measurements.

**Figure 6:**
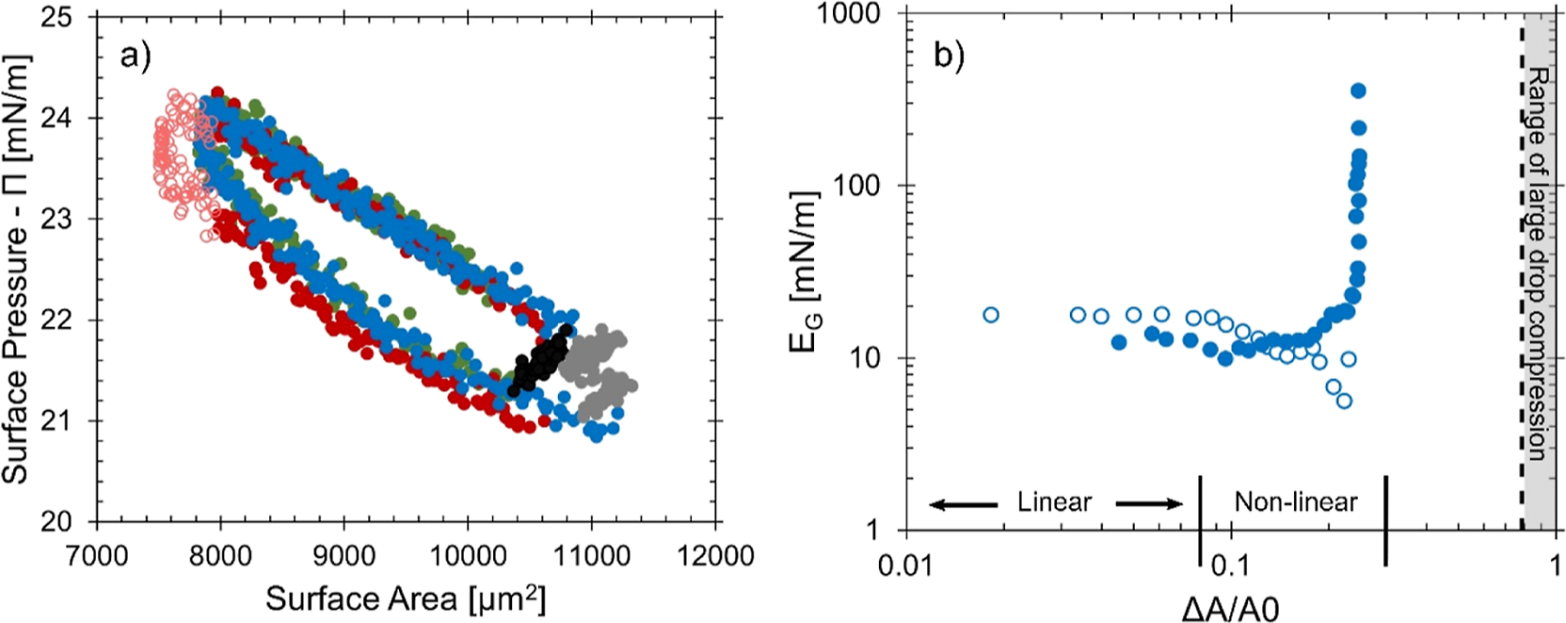
(a) Surface pressure (Π = γ – γ_0_, γ_0_ = 41 ± 1 mN/m) as a function of surface area for a single interface that has been subjected to three compression and expansions cycles (shown in the red, green, and blue symbols) The oil phase contained 1% C5 dispersed in isomatic 1–1, and interfaces were aged for 3 h prior to the compression cycles. The open symbols indicate area measurements where the curvature of the interface flattened and were not considered in further interfacial analysis. The black and gray symbols correspond to 10 s rest intervals between each cycle. (b) Gibbs modulus (*E*_G_) as a function of compression strain (Δ*A*/*A*_0_) for the first compression and expansion of a single interface. The open symbols show the Gibbs modulus during compression while the closed symbols show the modulus during expansion of the same interface. The gray shaded region shows the scale of compression strain required for interfacial buckling in the large pendant drop compressions.

Interfacial buckling is observed when the interface is subject to an applied strain of approximately 90%, and not with smaller linear deformations in the dilatational measurements. This suggests that adsorbed asphaltenes do not form a cross-linked network or solid-like film at the interface, even after long adsorption times. Instead, the rigidity of these asphaltene-laden interfaces is likely due to the jamming of larger, densely packed asphaltene aggregates that form in a poor solvent and adsorb to the o/w interface. This is in agreement with what has been observed in the studies of interfacial behavior of asphaltenes^31,50,79^ and the soft-glassy model in systems with low surface coverages below monolayer formation.^32,80^ To characterize the mechanics of jamming at asphaltene-laden interfaces in a poor solvent, nonlinear compressions and expansion cycles are applied to interfaces with 1 wt % C5 in isomatic 1–1 that has been aged for 3 h, which are conditions consistent with conditions where interfacial buckling was observed. Compressions are conducted using the microtensiometer platform by decreasing the pressure in the capillary-side fluid at a constant rate of ∼15 Pa s^–1^. Figure 6a shows the surface pressure response to three consecutive compression and expansion cycles on the same interface after 3 h of interfacial aging, with 10 s rest times between each cycle.

The maximum strain amplitude is 25% for this compression–expansion cycle. Hysteretic behavior in Figure 6a shows evidence of a relaxation time as the interface is compressed and re-expanded. This is shown to be reproducible over multiple cycles, suggesting that the interfacial structure changes due to the nonlinear compression and recovers back to its original state upon expansion. The Gibbs modulus,  taken from the local slope of the surface pressure and area measurements along the compression curve, is shown as a function of strain amplitude in Figure 6b. For this, the modulus is evaluated for the first compression (open symbols) followed by the immediate re-expansion (closed symbols) of a single interface.

At low strain amplitude (<10%), *E*_G_ is approximately 20 mN/m, which is on the same order as the dilatational modulus measured in both good and poor solvent conditions. In Figure 6b, nonlinear behavior in *E*_G_ is observed when compressed further with a strain of around 10%, as seen with the slight decrease in the modulus value, further suggesting changes to the interfacial structure due to this deformation. *E*_G_ increases by 1 order of magnitude upon expansion of the interface and returns to the same values at the end of the expansion process. The modulus behavior over the whole compression–expansion cycle could be an indication of interactions of adsorbed asphaltene aggregates and the start of interfacial jamming, before recovering to the same state. To fully characterize this complicated interfacial behavior associated with interfacial rigidity, the interface needs to be forced into a jammed state before the mechanics can be probed. In order to process the interface into a jammed state, poor solvent conditions, leading to the formation of larger asphaltene aggregates, and compression (high applied strain) are likely required. The interfacial mechanics in the good solvent are consistent with the study of emulsion stability by asphaltenes as well as the literature showing that emulsions are less stable with more aromatic content in the dispersion of the asphaltenes.^17,81^ Emulsion drops with these interfaces are likely to coalesce despite the decreased interfacial tension due to the low dilatational elasticity. Under dilatational deformation in both the good and poor solvent environments, interfaces were fluid-like when subject to low-strain compressions (<3.0%) and showed no indication of the formation of a connected interfacial network and rigid film. However, measurements from interfaces under shear deformation allude to a solid-like network of asphaltenes when dispersed in a poor solvent environment.^33^ These results show that dilatational and shear stresses can result in different deformation properties of asphaltene-laden interfaces. Care must be taken when only one rheological characterization is utilized to study the interfacial network.

Rigidified interfaces in the literature have been observed in systems where two adjacent drops have been ruptured and forced to coalesce,^82^ or after large compressions were imposed on interfaces until buckling and failure of the interface was observed.^14,23^ In both cases, interfaces are forced into this state due to the areal change during deformation. The available interfacial area is decreased. With no indication of high interfacial elasticity under low-strain dilatational deformations it is unlikely that the formation of a cross-linked interfacial framework gives rise to interfacial buckling associated with the observed rigidified interfaces. This suggests that the large areal changes from these high strain deformations lead to the jamming of asphaltene aggregates at the interface, leading to the observed rigid behavior. Emulsion droplets in this jammed state are less likely to coalesce due to the maximum packing of adsorbed solid material at the interface.

By quantifying interfacial behavior, this data set provides a benchmark for characterizing asphaltene systems, where the coated interfaces are not stable against the coalescence. Additionally, conditions required for emulsion droplets to form mechanically rigid interfaces are identified. Within isomatic 1–4 (good solvent condition), the maximum surface concentration of asphaltenes is about 20% of that in a poor solvent condition reported in the literature.^31^ The higher surface pressures measured with asphaltenes dispersed in isomatic 1–1 also suggest a higher maximum surface coverage due to the unfavorable solvent environment. The concentration regime of this work is relevant to all colloidal forms of asphaltenes under a good solvent system. The average size of asphaltene molecules, nanoaggregates, and clusters of nanoaggregates, defined in the Yen–Mullins model, are typically 1.5, 2.0, and 5.0 nm, respectively. Despite the increase in size due to associations, the asphaltene nanoaggregates and clusters are still stable on a colloidal level in the good solvent, demonstrated by the millifluidic experiment.^65^ The bulk concentrations used for this work are assumed to be near or above the concentration at which clusters do not undergo further growth, defined as the critical cluster concentration. Therefore, it is expected that adsorbed asphaltenes at the o/w interface would primarily be composed of these clusters under good solvent conditions. In the poor solvent, as the asphaltene clusters are destabilized and aggregate further, the size of the asphaltene aggregates adsorbing to the o/w interface is expected to be larger under the poor solvent conditions. Work done in a study by Spiecker et al., used SANS measurements of Heptol systems to measure the structure of asphaltene aggregates as a function of solvent composition.^3,47^ Results from these studies show that the particle size of asphaltene at an interfacial film was on the order of 25 nm or greater^47^ and aggregates that precipitate out of the bulk in aliphatic-rich solvent environments can be as large as 100 nm.^3^ Therefore, it is likely that larger asphaltene structures are adsorbing to the asphaltene-coated interface, leading to the interfacial jamming of asphaltene solids under large compressions.

## Conclusions

Interfacial properties of asphaltenes at the o/w interface are characterized under good and poor solvent conditions. Under good solvent conditions, rich in aromatic solvent, the interface remains fluid-like. In contrast, poor solvent conditions lead to interfacial jamming, associated with rigid interfaces observed at asphaltene-laden interfaces under high-amplitude compressions. In an aromatic-rich environment, the system consists primarily of well-dispersed nanoaggregates and clusters, while the poor solvent conditions lead to further assembly of larger aggregates. Under both solvent conditions and aggregation states, asphaltenes are shown to be interfacially active. Measurements of surface pressure and low-amplitude dilatational modulus will not indicate if an interface will jam and are not indicators of the overall interfacial structure. The solvent quality of the system, concentration of bulk asphaltenes, and age of the interface all contribute to whether adsorbed asphaltenes will jam at the o/w interface. Under the right conditions, jamming of asphaltene-laden interfaces can be seen with large amplitude compressions of approximately 90% strain. This data provides information relevant to model development and application for asphaltene-coated o/w interfaces.

## Supporting Information Available

Schematic of components for the microbutton equipment, effects of dilatational measurements on interfacial tension, and additional experimental measurements of creep compliance tested at different torque values (PDF)

## References

[ref1] Mullins O. C. The Modified Yen Model. Energy Fuels 2010, 24 (4), 2179–2207. 10.1021/ef900975e.

[ref2] Zuo J. Y.; Elshahawi H.; Mullins O. C.; Dong C.; Zhang D.; Jia N.; Zhao H. Asphaltene Gradients and Tar Mat Formation in Reservoirs under Active Gas Charging. Fluid Phase Equilib. 2012, 315, 91–98. 10.1016/j.fluid.2011.11.024.

[ref3] Spiecker P. M.; Gawrys K. L.; Kilpatrick P. K. Aggregation and Solubility Behavior of Asphaltenes and Their Subfractions. J. Colloid Interface Sci. 2003, 267 (1), 178–193. 10.1016/S0021-9797(03)00641-6. 14554184

[ref4] Turgman-Cohen S.; Smith M. B.; Fischer D. A.; Kilpatrick P. K.; Genzer J. Asphaltene Adsorption onto Self-Assembled Monolayers of Mixed Aromatic and Aliphatic Trichlorosilanes. Langmuir 2009, 25 (11), 6260–6269. 10.1021/la9000895. 19334746

[ref5] Mullins O. C.; Sabbah H.; Eyssautier J.; Pomerantz A. E.; Barré L.; Andrews A. B.; Ruiz-Morales Y.; Mostowfi F.; McFarlane R.; Goual L.; Lepkowicz R.; Cooper T.; Orbulescu J.; Leblanc R. M.; Edwards J.; Zare R. N. Advances in Asphaltene Science and the Yen-Mullins Model. Energy Fuels 2012, 26 (7), 3986–4003. 10.1021/ef300185p.

[ref6] Schuler B.; Zhang Y.; Liu F.; Pomerantz A. E.; Andrews A. B.; Gross L.; Pauchard V.; Banerjee S.; Mullins O. C. Overview of Asphaltene Nanostructures and Thermodynamic Applications. Energy Fuels 2020, 34 (12), 15082–15105. 10.1021/acs.energyfuels.0c00874.

[ref7] Zuo J. Y.; Mullins O. C.; Freed D.; Elshahawi H.; Dong C.; Seifert D. J. Advances in the Flory-Huggins-Zuo Equation of State for Asphaltene Gradients and Formation Evaluation. Energy Fuels 2013, 27 (4), 1722–1735. 10.1021/ef301239h.

[ref8] Gray M. R.; Yarranton H. W.; Chacón-Patiño M. L.; Rodgers R. P.; Bouyssiere B.; Giusti P. Distributed Properties of Asphaltene Nanoaggregates in Crude Oils: A Review. Energy Fuels 2021, 35 (22), 18078–18103. 10.1021/acs.energyfuels.1c01837.

[ref9] Zuo J. Y.; Mullins O. C.; Mishra V.; Garcia G.; Dong C.; Zhang D. Asphaltene Grading and Tar Mats in Oil Reservoirs. Energy Fuels 2012, 26 (3), 1670–1680. 10.1021/ef201218m.

[ref10] McLean J. D.; Kilpatrick P. K. Effects of Asphaltene Aggregation in Model Heptane-Toluene Mixtures on Stability of Water-in-Oil Emulsions. J. Colloid Interface Sci. 1997, 196 (1), 23–34. 10.1006/jcis.1997.5177. 9441646

[ref11] Gawrys K. L.; Kilpatrick P. K. Asphaltene Aggregation: Techniques for Analysis. Instrum. Sci. Technol. 2004, 32 (3), 247–253. 10.1081/CI-120030536.

[ref12] Verruto V. J.; Kilpatrick P. K. Preferential Solvent Partitioning within Asphaltenic Aggregates Dissolved in Binary Solvent Mixtures. Energy Fuels 2007, 21 (3), 1217–1225. 10.1021/ef060456n.

[ref13] Nenningsland A. L.; Simon S.; Sjöblom J. Influence of Interfacial Rheological Properties on Stability of Asphaltene-Stabilized Emulsions. J. Dispersion Sci. Technol. 2014, 35 (2), 231–243. 10.1080/01932691.2013.784196.

[ref14] Jeribi M.; Almir-Assad B.; Langevin D.; Hénaut I.; Argillier J. F. Adsorption Kinetics of Asphaltenes at Liquid Interfaces. J. Colloid Interface Sci. 2002, 256 (2), 268–272. 10.1006/jcis.2002.8660.

[ref15] Quintero C. G.; Noïk C.; Dalmazzone C.; Grossiord J. L. Formation Kinetics and Viscoelastic Properties of Water/Crude Oil Interfacial Films. Oil Gas Sci. Technol.—Rev. l’IFP 2009, 64 (5), 607–616. 10.2516/ogst/2009031.

[ref16] Verruto V. J.; Le R. K.; Kilpatrick P. K. Adsorption and Molecular Rearrangement of Amphoteric Species at Oil-Water Interfaces. J. Phys. Chem. B 2009, 113 (42), 13788–13799. 10.1021/jp902923j. 19583194

[ref17] McLean J. D.; Kilpatrick P. K. Effects of Asphaltene Solvency on Stability of Water-in-Crude-Oil Emulsions. J. Colloid Interface Sci. 1997, 189 (2), 242–253. 10.1006/jcis.1997.4807. 9441646

[ref18] Rane J. P.; Harbottle D.; Pauchard V.; Couzis A.; Banerjee S. Adsorption Kinetics of Asphaltenes at the Oil-Water Interface and Nanoaggregation in the Bulk. Langmuir 2012, 28 (26), 9986–9995. 10.1021/la301423c. 22680071

[ref19] Jian C.; Poopari M. R.; Liu Q.; Zerpa N.; Zeng H.; Tang T. Reduction of Water/Oil Interfacial Tension by Model Asphaltenes: The Governing Role of Surface Concentration. J. Phys. Chem. B 2016, 120 (25), 5646–5654. 10.1021/acs.jpcb.6b03691. 27268710

[ref20] Morais W. J. S.; Franceschi E.; Dariva C.; Borges G. R.; Santos A. F.; Santana C. C. Dilatational Rheological Properties of Asphaltenes in Oil-Water Interfaces: Langmuir Isotherm and Influence of Time, Concentration, and Heptol Ratios. Energy Fuels 2017, 31 (9), 10233–10244. 10.1021/acs.energyfuels.7b01633.

[ref21] Yeung A.; Dabros T.; Czarnecki J.; Masliyah J. On the interfacial properties of micrometre–sized water droplets in crude oil. Proc. R. Soc. London, A 1999, 455, 3709–3723. 10.1098/rspa.1999.0473.

[ref22] Wu X. Investigating the Stability Mechanism of Water-in-Diluted Bitumen Emulsions through Isolation and Characterization of the Stabilizing Materials at the Interface. Energy Fuels 2003, 17 (1), 179–190. 10.1021/ef020098y.

[ref23] Kimbler O. K.; Reed R. L.; Silberberg I. H. Physical Characteristics of Natural Films Formed at Crude Oil-Water Interfaces. Soc. Pet. Eng. J. 1966, 6 (02), 153–165. 10.2118/1201-PA.

[ref24] Pradilla D.; Simon S.; Sjöblom J.; Samaniuk J.; Skrzypiec M.; Vermant J. Sorption and Interfacial Rheology Study of Model Asphaltene Compounds. Langmuir 2016, 32 (12), 2900–2911. 10.1021/acs.langmuir.6b00195. 26949974

[ref25] Qiao P.; Harbottle D.; Tchoukov P.; Wang X.; Xu Z. Asphaltene Subfractions Responsible for Stabilizing Water-in-Crude Oil Emulsions. Part 3. Effect of Solvent Aromaticity. Energy Fuels 2017, 31 (9), 9179–9187. 10.1021/acs.energyfuels.7b01387.

[ref26] Goodarzi F.; Zendehboudi S. A Comprehensive Review on Emulsions and Emulsion Stability in Chemical and Energy Industries. Can. J. Chem. Eng. 2019, 97 (1), 281–309. 10.1002/cjce.23336.

[ref27] Sheu E. Y. Petroleum AsphalteneProperties, Characterization, and Issues. Energy Fuels 2002, 16 (1), 74–82. 10.1021/ef010160b.

[ref28] Fakher S.; Ahdaya M.; Elturki M.; Imqam A. Critical Review of Asphaltene Properties and Factors Impacting Its Stability in Crude Oil. J. Pet. Explor. Prod. Technol. 2020, 10 (3), 1183–1200. 10.1007/s13202-019-00811-5.

[ref29] Moghadasi R.; Kord S.; Moghadasi J.; Dashti H. Mechanistic Understanding of Asphaltenes Surface Behavior at Oil/Water Interface: An Experimental Study. J. Mol. Liq. 2019, 285, 562–571. 10.1016/j.molliq.2019.04.123.

[ref30] Schuler B.; Meyer G.; Peña D.; Mullins O. C.; Gross L. Unraveling the Molecular Structures of Asphaltenes by Atomic Force Microscopy. J. Am. Chem. Soc. 2015, 137 (31), 9870–9876. 10.1021/jacs.5b04056. 26170086

[ref31] Pauchard V.; Rane J. P.; Banerjee S. Asphaltene-Laden Interfaces Form Soft Glassy Layers in Contraction Experiments: A Mechanism for Coalescence Blocking. Langmuir 2014, 30 (43), 12795–12803. 10.1021/la5028042. 25330092

[ref32] Samaniuk J. R.; Hermans E.; Verwijlen T.; Pauchard V.; Vermant J. Soft-Glassy Rheology of Asphaltenes at Liquid Interfaces. J. Dispersion Sci. Technol. 2015, 36 (10), 1444–1451. 10.1080/01932691.2015.1022654.

[ref33] Chang C.-C.; Nowbahar A.; Mansard V.; Williams I.; Mecca J.; Schmitt A. K.; Kalantar T. H.; Kuo T.-C.; Squires T. M. Interfacial Rheology and Heterogeneity of Aging Asphaltene Layers at the Water-Oil Interface. Langmuir 2018, 34 (19), 5409–5415. 10.1021/acs.langmuir.8b00176. 29685033

[ref34] Ruiz-Morales Y.; Alvarez-Ramírez F. Mesoscale Dissipative Particle Dynamics to Investigate Oil Asphaltenes and Sodium Naphthenates at the Oil-Water Interface. Energy Fuels 2021, 35 (11), 9294–9311. 10.1021/acs.energyfuels.1c00589.

[ref35] Ruiz-Morales Y.; Mullins O. C. Coarse-Grained Molecular Simulations to Investigate Asphaltenes at the Oil-Water Interface. Energy Fuels 2015, 29 (3), 1597–1609. 10.1021/ef502766v.

[ref36] Gao F.; Xu Z.; Liu G.; Yuan S. Molecular Dynamics Simulation: The Behavior of Asphaltene in Crude Oil and at the Oil/Water Interface. Energy Fuels 2014, 28 (12), 7368–7376. 10.1021/ef5020428.

[ref37] Verruto V. J.; Kilpatrick P. K. Water-in-Model Oil Emulsions Studied by Small-Angle Neutron Scattering: Interfacial Film Thickness and Composition. Langmuir 2008, 24 (22), 12807–12822. 10.1021/la802095m. 18947210

[ref38] Jestin J.; Simon S.; Zupancic L.; Barré L. A Small Angle Neutron Scattering Study of the Adsorbed Asphaltene Layer in Water-in-Hydrocarbon Emulsions: Structural Description Related to Stability. Langmuir 2007, 23 (21), 10471–10478. 10.1021/la701193f. 17867712

[ref39] Alvarez G.; Jestin J.; Argillier J. F.; Langevin D. Small-Angle Neutron Scattering Study of Crude Oil Emulsions: Structure of the Oil-Water Interfaces. Langmuir 2009, 25 (7), 3985–3990. 10.1021/la802736c. 19714887

[ref40] Kilpatrick P. K. Water-in-Crude Oil Emulsion Stabilization: Review and Unanswered Questions. Energy Fuels 2012, 26 (7), 4017–4026. 10.1021/ef3003262.

[ref41] Czarnecki J.; Tchoukov P.; Dabros T.; Xu Z. Role of Asphaltenes in Stabilisation of Water in Crude Oil Emulsions. Can. J. Chem. Eng. 2013, 91 (8), 1365–1371. 10.1002/cjce.21835.

[ref42] Czarnecki J.; Moran K. On the Stabilization Mechanism of Water-in-Oil Emulsions in Petroleum Systems. Energy Fuels 2005, 19 (5), 2074–2079. 10.1021/ef0501400.

[ref43] McLean J. D.; Kilpatrick P. K. Effects of Asphaltene Solvency on Stability of Water-in-Crude-Oil Emulsions. J. Colloid Interface Sci. 1997, 189, 242–253. 10.1006/jcis.1997.4807. 9441646

[ref44] Yudin I. K.; Nikolaenko G. L.; Gorodetskii E. E.; Markhashov E. L.; Frot D.; Briolant Y.; Agayan V. A.; Anisimov M. A. Universal Behavior of Asphaltene Aggregation in Hydrocarbon Solutions. Pet. Sci. Technol. 1998, 16 (3–4), 395–414. 10.1080/10916469808949790.

[ref45] Yang X.; Verruto V. J.; Kilpatrick P. K. Dynamic Asphaltene-Resin Exchange at the Oil/Water Interface: Time-Dependent W/O Emulsion Stability for Asphaltene/Resin Model Oils. Energy Fuels 2007, 21 (3), 1343–1349. 10.1021/ef060465w.

[ref46] Rane J. P.; Pauchard V.; Couzis A.; Banerjee S. Interfacial Rheology of Asphaltenes at Oil-Water Interfaces and Interpretation of the Equation of State. Langmuir 2013, 29 (15), 4750–4759. 10.1021/la304873n. 23506138

[ref47] Spiecker P. M.; Kilpatrick P. K. Interfacial Rheology of Petroleum Asphaltenes at the Oil-Water Interface. Langmuir 2004, 20 (10), 4022–4032. 10.1021/la0356351. 15969394

[ref48] Harbottle D.; Chen Q.; Moorthy K.; Wang L.; Xu S.; Liu Q.; Sjoblom J.; Xu Z. Problematic Stabilizing Films in Petroleum Emulsions: Shear Rheological Response of Viscoelastic Asphaltene Films and the Effect on Drop Coalescence. Langmuir 2014, 30 (23), 6730–6738. 10.1021/la5012764. 24845467

[ref49] Schuler B.; Fatayer S.; Meyer G.; Rogel E.; Moir M.; Zhang Y.; Harper M. R.; Pomerantz A. E.; Bake K. D.; Witt M.; Peña D.; Kushnerick J. D.; Mullins O. C.; Ovalles C.; Van Den Berg F. G. A.; Gross L. Heavy Oil Based Mixtures of Different Origins and Treatments Studied by Atomic Force Microscopy. Energy Fuels 2017, 31 (7), 6856–6861. 10.1021/acs.energyfuels.7b00805.

[ref50] Bridot J. L.; Langevin D.; Mullins O. C. Role of Asphaltene Origin in Its Adsorption at Oil-Water Interfaces. Energy Fuels 2022, 36 (16), 8749–8759. 10.1021/acs.energyfuels.2c00966.

[ref51] Klein G. C.; Kim S.; Rodgers R. P.; Marshall A. G.; Yen A. Mass Spectral Analysis of Asphaltenes. II. Detailed Compositional Comparison of Asphaltenes Deposit to Its Crude Oil Counterpart for Two Geographically Different Crude Oils by ESI FT-ICR MS. Energy Fuels 2006, 20 (5), 1973–1979. 10.1021/ef0600208.

[ref52] Fossen M.; Kallevik H.; Knudsen K. D.; Sjöblom J. Asphaltenes Precipitated by a Two-Step Precipitation Procedure. 2. Physical and Chemical Characteristics. Energy Fuels 2011, 25 (8), 3552–3567. 10.1021/ef200373v.

[ref53] Ruiz-Morales Y. Application of the Y-Rule and Theoretical Study to Understand the Topological and Electronic Structures of Polycyclic Aromatic Hydrocarbons from Atomic Force Microscopy Images of Soot, Coal Asphaltenes, and Petroleum Asphaltenes. Energy Fuels 2022, 36 (16), 8725–8748. 10.1021/acs.energyfuels.2c01170.

[ref54] Chacón-Patiño M. L.; Rowland S. M.; Rodgers R. P. Advances in Asphaltene Petroleomics. Part 3. Dominance of Island or Archipelago Structural Motif Is Sample Dependent. Energy Fuels 2018, 32 (9), 9106–9120. 10.1021/acs.energyfuels.8b01765.

[ref55] Sharma A.; Groenzin H.; Tomita A.; Mullins O. C. Probing Order in Asphaltenes and Aromatic Ring Systems by HRTEM. Energy Fuels 2002, 16 (2), 490–496. 10.1021/ef010240f.

[ref56] Liu D.; Li C.; Zhang X.; Yang F.; Sun G.; Yao B.; Zhang H. Polarity Effects of Asphaltene Subfractions on the Stability and Interfacial Properties of Water-in-Model Oil Emulsions. Fuel 2020, 269 (June 2019), 117450 10.1016/j.fuel.2020.117450.

[ref57] Dicharry C.; Arla D.; Sinquin A.; Graciaa A.; Bouriat P. Stability of Water/Crude Oil Emulsions Based on Interfacial Dilatational Rheology. J. Colloid Interface Sci. 2006, 297 (2), 785–791. 10.1016/j.jcis.2005.10.069. 16324706

[ref58] Hou J.; Feng X.; Masliyah J.; Xu Z. Understanding Interfacial Behavior of Ethylcellulose at the Water-Diluted Bitumen Interface. Energy Fuels 2012, 26 (3), 1740–1745. 10.1021/ef201722y.

[ref59] Nowbahar A.; Whitaker K. A.; Schmitt A. K.; Kuo T. C. Mechanistic Study of Water Droplet Coalescence and Flocculation in Diluted Bitumen Emulsions with Additives Using Microfluidics. Energy Fuels 2017, 31 (10), 10555–10565. 10.1021/acs.energyfuels.7b01619.

[ref60] Moran K.; Yeung A.; Masliyah J. Measuring Interfacial Tensions of Micrometer-Sized Droplets: A Novel Micromechanical Technique. Langmuir 1999, 15 (24), 8497–8504. 10.1021/la990363g.

[ref61] Rodríguez-Hakim M.; Anand S.; Tajuelo J.; Yao Z.; Kannan A.; Fuller G. G. Asphaltene-Induced Spontaneous Emulsification: Effects of Interfacial Co-Adsorption and Viscoelasticity. J. Rheol. 2020, 64 (4), 799–816. 10.1122/1.5145307.

[ref62] Bochner De Araujo S.; Merola M.; Vlassopoulos D.; Fuller G. G. Droplet Coalescence and Spontaneous Emulsification in the Presence of Asphaltene Adsorption. Langmuir 2017, 33 (40), 10501–10510. 10.1021/acs.langmuir.7b02638. 28889742

[ref63] Azari V.; Abolghasemi E.; Hosseini A.; Ayatollahi S.; Dehghani F. Electrokinetic Properties of Asphaltene Colloidal Particles: Determining the Electric Charge Using Micro Electrophoresis Technique. Colloids Surf., A 2018, 541 (November 2017), 68–77. 10.1016/j.colsurfa.2018.01.029.

[ref64] Huh C.; Scriven L. E. Hydrodynamic Model of Steady Movement of a Solid/Liquid/Fluid Contact Line. J. Colloid Interface Sci. 1971, 35 (1), 85–101. 10.1016/0021-9797(71)90188-3.

[ref65] Haider O. M.; Ma J.; Grzesiak K. A.; Walker L. M. Advantages of a Millifluidic Approach to the Characterization of Long-Time Solvency Effects on Bulk Asphaltene Precipitation. Energy Fuels 2023, 37, 2791–2798. 10.1021/acs.energyfuels.2c03918.

[ref66] Bleier B. J.; Yezer B. A.; Freireich B. J.; Anna S. L.; Walker L. M. Droplet-Based Characterization of Surfactant Efficacy in Colloidal Stabilization of Carbon Black in Nonpolar Solvents. J. Colloid Interface Sci. 2017, 493, 265–274. 10.1016/j.jcis.2017.01.033. 28110061

[ref67] Alvarez N. J.; Walker L. M.; Anna S. L. A Microtensiometer To Probe the Effect of Radius of Curvature on Surfactant Transport to a Spherical Interface. Langmuir 2010, 26 (16), 13310–13319. 10.1021/la101870m. 20695573

[ref68] Reichert M. D.; Alvarez N. J.; Brooks C. F.; Grillet A. M.; Mondy L. A.; Anna S. L.; Walker L. M. The Importance of Experimental Design on Measurement of Dynamic Interfacial Tension and Interfacial Rheology in Diffusion-Limited Surfactant Systems. Colloids Surf., A 2015, 467, 135–142. 10.1016/j.colsurfa.2014.11.035.

[ref69] Alvarez N. J.; Walker L. M.; Anna S. L. A Criterion to Assess the Impact of Confined Volumes on Surfactant Transport to Liquid-Fluid Interfaces. Soft Matter 2012, 8 (34), 8917–8925. 10.1039/c2sm25447f.

[ref70] Davidson M. L.; Walker L. M. Interfacial Properties of Polyelectrolyte-Surfactant Aggregates at Air/Water Interfaces. Langmuir 2018, 34 (43), 12906–12913. 10.1021/acs.langmuir.8b02438. 30274519

[ref71] Kirby S. M.; Anna S. L.; Walker L. M. Effect of Surfactant Tail Length and Ionic Strength on the Interfacial Properties of Nanoparticle-Surfactant Complexes. Soft Matter 2018, 14 (1), 112–123. 10.1039/C7SM01806A. 29214259

[ref72] Kirby S. M.; Anna S. L.; Walker L. M. Sequential Adsorption of an Irreversibly Adsorbed Nonionic Surfactant and an Anionic Surfactant at an Oil/Aqueous Interface. Langmuir 2015, 31 (14), 4063–4071. 10.1021/la504969v. 25798716

[ref73] Kirby S. M.; Zhang X.; Russo P. S.; Anna S. L.; Walker L. M. Formation of a Rigid Hydrophobin Film and Disruption by an Anionic Surfactant at an Air/Water Interface. Langmuir 2016, 32 (22), 5542–5551. 10.1021/acs.langmuir.6b00809. 27164189

[ref74] Barman S.; Davidson M. L.; Walker L. M.; Anna S. L.; Zasadzinski J. A. Inflammation Product Effects on Dilatational Mechanics Can Trigger the Laplace Instability and Acute Respiratory Distress Syndrome. Soft Matter 2020, 16 (29), 6890–6901. 10.1039/D0SM00415D. 32643749 PMC7462632

[ref75] Davidson M. L.; Laufer L.; Gottlieb M.; Walker L. M. Transport of Flexible, Oil-Soluble Diblock and BAB Triblock Copolymers to Oil/Water Interfaces. Langmuir 2020, 36 (26), 7227–7235. 10.1021/acs.langmuir.0c00477. 32482075

[ref76] Choi S. Q.; Jang S. G.; Pascall A. J.; Dimitriou M. D.; Kang T.; Hawker C. J.; Squires T. M. Synthesis of Multifunctional Micrometer-Sized Particles with Magnetic, Amphiphilic, and Anisotropic Properties. Adv. Mater. 2011, 23 (20), 2348–2352. 10.1002/adma.201003604. 21360773

[ref77] Anisimov M. A.; Yudin I. K.; Nikitin V.; Nikolaenko G.; Chernoutsan A.; Toulhoat H.; Frot D.; Briolant Y. Asphaltene Aggregation in Hydrocarbon Solutions Studied by Photon Correlation Spectroscopy. J. Phys. Chem. 1995, 99 (23), 9576–9580. 10.1021/j100023a040.

[ref78] Langevin D. Rheology of Adsorbed Surfactant Monolayers at Fluid Surfaces. Annu. Rev. Fluid. Mech. 2014, 46 (1), 47–65. 10.1146/annurev-fluid-010313-141403.

[ref79] Pauchard V.; Roy T. Blockage of Coalescence of Water Droplets in Asphaltenes Solutions: A Jamming Perspective. Colloids Surf., A 2014, 443, 410–417. 10.1016/j.colsurfa.2013.12.001.

[ref80] Alicke A.; Simon S.; Sjöblom J.; Vermant J. Assessing the Interfacial Activity of Insoluble Asphaltene Layers: Interfacial Rheology versus Interfacial Tension. Langmuir 2020, 36 (49), 14942–14959. 10.1021/acs.langmuir.0c02234. 33264021

[ref81] Fan Y.; Simon S.; Sjöblom J. Interfacial Shear Rheology of Asphaltenes at Oil-Water Interface and Its Relation to Emulsion Stability: Influence of Concentration, Solvent Aromaticity and Nonionic Surfactant. Colloids Surf., A 2010, 366 (1–3), 120–128. 10.1016/j.colsurfa.2010.05.034.

[ref82] Strassner J. E. Effect of PH on Interfacial Films and Stability of Crude Oil-Water Emulsions. J. Pet. Technol. 1968, 20 (03), 303–312. 10.2118/1939-PA.

