# Supplementary material for: Solvent Quality and Aggregation State of Asphaltenes on Interfacial Mechanics and Jamming Behavior at the Oil/Water Interface
Source: Langmuir. 2023 Oct 20;39(43):15238–48. doi: 10.1021/acs.langmuir.3c01890 (PMC10620990; doi:10.1021/acs.langmuir.3c01890)
Supplement: Supplementary file 1 — la3c01890_si_001.pdf [file la3c01890_si_001.pdf]

Supporting Information – Solvent quality and aggregation state of asphaltenes on interfacial mechanics and jamming behavior at the oil/water interface

Junchi Ma[†,1], Olivia M. Haider[†,1], Chih-Cheng Chang[2], Kathryn A. Grzesiak[3], Todd M. Squires[2], and Lynn M. Walker[*,1]

[1] Department of Chemical Engineering, Carnegie Mellon University, Pittsburgh, Pennsylvania, 15213, United States

[2] Department of Chemical Engineering, University of California, Santa Barbara, California, 93106, United States

[3] The Dow Chemical Company, Midland, Michigan, 48640, United States

[†]shared first authorship

*corresponding author: lwalker@andrew.cmu.edu

Details:

This supporting information contains 4 pages and 3 figures.

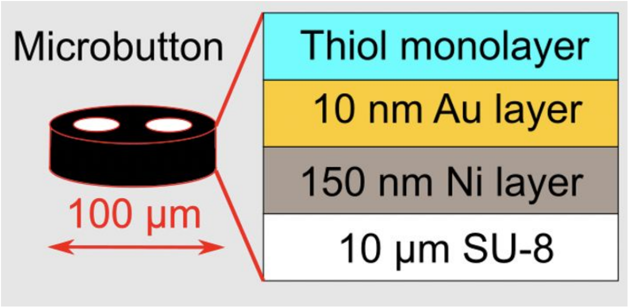

Figure S1: The 100 µm microbutton is composed of 10 µm SU-8 photoresist as hydrophobic side, 150 nm Ni layer as ferromagnetic layer and 10 nm Au layer to anchor the thiol monolayer for hydrophilic side.

The design of the microbutton is detailed in Figure S1. A 10 µm-thick SU-8 photoresist forms the hydrophobic side of the microbutton. A 150 nm nickel layer is deposited on the SU-8 layer to provide ferromagnet to the microbutton. A 10 nm gold layer is then deposited on the nickel layer to anchor the thiol monolayer in order to form a hydrophilic surface on the top of the microbutton.

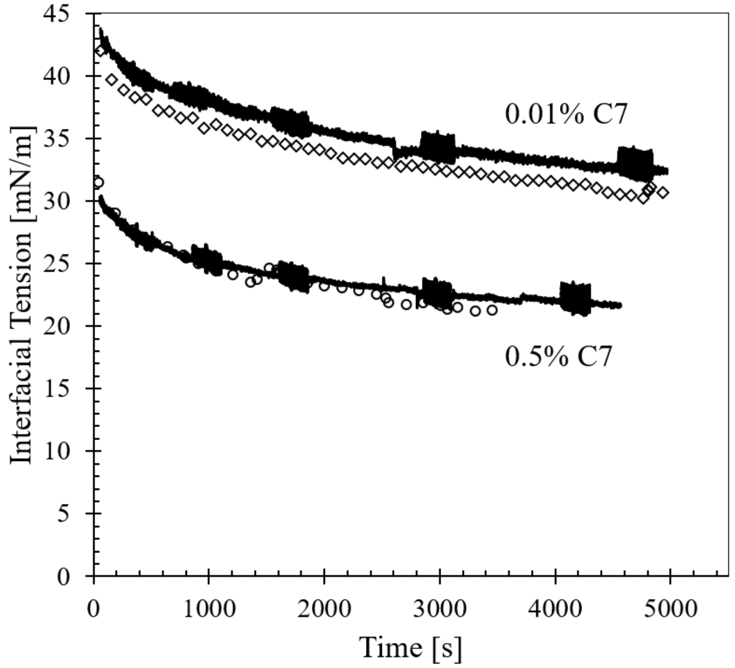

Figure S2: Dynamic interfacial tension while measuring dilatational rheology at different time. Empty symbols represent the dynamic interfacial tension of 0.01% asphaltene C7 (diamonds) and 0.5% asphaltene C7 (circles). Lines show the dilatational measurements as 0.01% asphaltene C7 (top) and 0.5% asphaltene C7 (bottom) adsorb to the interface.

Figure S2 shows the comparison of adsorption dynamics of two asphaltene C7 concentrations with (lines) and without (diamonds and circles) transient dilatational measurements. For 0.01% asphaltene C7, dynamic interfacial tension with dilatational measurements is slightly higher due to a lower initial curvature of oil-water interface than the one without dilatation. Overall, performing dilatational rheology at different time points has minimal impact on adsorption dynamics.

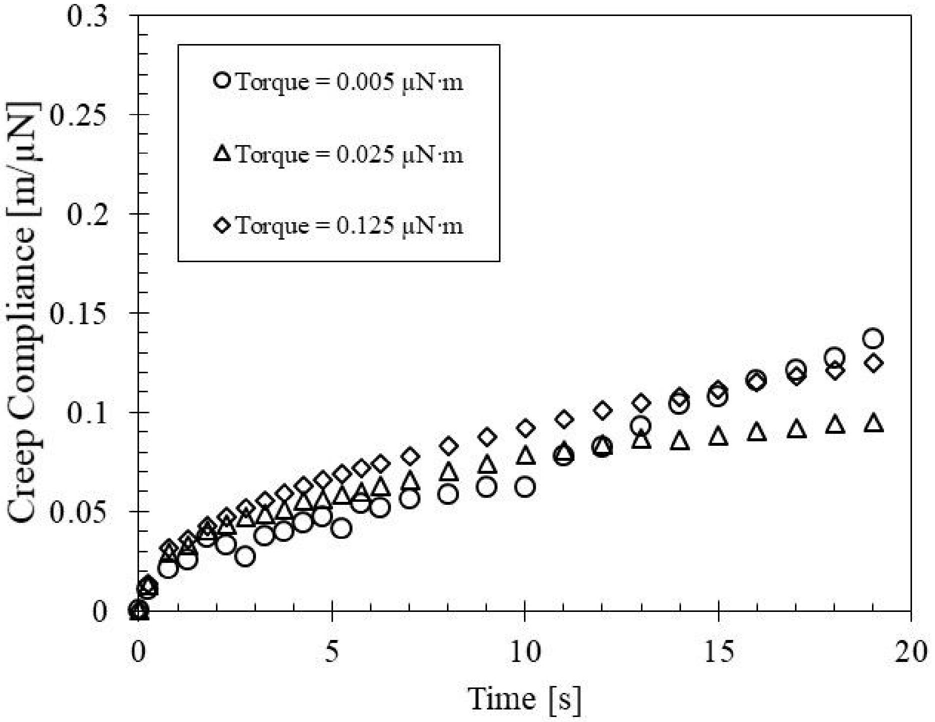

Figure S3 Creep compliance for the asphaltene C7 interfacial layer using DWR. Circles: torque = 0.005 μN·m; Triangles: torque = 0.025 μN·m; Diamonds: torque = 0.125 μN·m.

Figure S3 shows the creep compliance of the asphaltene C7 adsorbed layer at the oil-water interface at three different torques. Before the measurement, the interface has aged for 3000 seconds. For these three torques tested, the creep compliance as a function of time is consistent so that a linear torque region is found for the creep compliance measurement.
